# Processed and Unprocessed Red Meat Consumption and Risk for Type 2 Diabetes Mellitus: An Updated Meta-Analysis of Cohort Studies

**DOI:** 10.3390/ijerph182010788

**Published:** 2021-10-14

**Authors:** Rui Zhang, Jialin Fu, Justin B. Moore, Lee Stoner, Rui Li

**Affiliations:** 1 College of Life Sciences, South-Central University for Nationalities, Wuhan 430074, China; zhangrui@mail.scuec.edu.cn; 2 Department of Healthcare Management, School of Health Sciences, Wuhan University, Wuhan 430071, China; Fjl0708@whu.edu.cn; 3 Department of Implementation Science, Division of Public Health Sciences, Wake Forest School of Medicine, Winston-Salem, NC 27101, USA; jusmoore@wakehealth.edu; 4 Department of Epidemiology & Prevention, Division of Public Health Sciences, Wake Forest School of Medicine, Winston-Salem, NC 27101, USA; 5 Department of Family & Community Medicine, Wake Forest School of Medicine, Winston-Salem, NC 27101, USA; 6 Department of Exercise & Sport Science, University of North Carolina, Chapel Hill, NC 27101, USA; stonerl@email.unc.edu

**Keywords:** processed red meat, unprocessed red meat, type 2 diabetes mellitus

## Abstract

Type II diabetes mellitus (T2DM) is a metabolic disorder that occurs in the body because of decreased insulin activity and/or insulin secretion. The incidence of T2DM has rapidly increased over recent decades. The relation between consumption of different types of red meats and risk of T2DM remains uncertain. This meta-analysis was conducted to quantitatively assess the associations of processed red meat (PRM) and unprocessed red meat (URM) consumption with T2DM. We searched PubMed, Embase, Web of Science and The Cochrane Library for English-language cohort studies published before January 2021. Summary relative risks (RR) with 95% confidence interval (CI) were estimated using fixed effects and random effects. Additionally, dose–response relationships were explored using meta-regression. Fifteen studies (*n* = 682,963 participants, cases = 50,675) were identified. Compared with the lowest intake group, high consumption of PRM and URM increased T2DM risk by 27% (95% CI 1.15–1.40) and 15% (95% CI 1.08–1.23), respectively. These relationships were consistently strongest for U.S-based studies, though the effects of sex are inconclusive. In conclusion, PRM and URM are both positively associated with T2DM incidence, and these relationships are strongest in the U.S. reduction of red meat consumption should be explored as a target for T2DM prevention initiatives.

## 1. Introduction

Type II diabetes mellitus (T2DM) is a serious, chronic disease that occurs either when the pancreas does not produce enough insulin (a hormone that regulates blood glucose), or when the body cannot effectively use the insulin it produces [1]. The global prevalence of T2DM has increased in recent decades. It was estimated that there were 451 million patients with T2DM worldwide in 2017, and this number is expected to increase to 693 million by 2045 [2]. Factors likely contributing to the increased prevalence of T2DM include improved rates of survival and enhanced detection, as well as increases in modifiable factors such as obesity, physical inactivity and poor dietary intake [3,4]. One common dietary component, which is an important source of protein across countries and cultures, is red meat. Red meat is defined as all types of mammalian muscle meat, such as beef, lamb, pork and game, and it can be divided into processed (PRM) and unprocessed (URM) red meat according to whether it is processed to extend its shelf life by curing, smoking, salting or the addition of chemical preservatives [5].

Some studies have reported PRM or URM consumption to be positively associated with T2DM incidence [6,7,8,9,10,11,12,13,14,15,16]. However, other studies have reported an inverse or null association between red meat intake and T2DM incidence [17,18,19,20,21,22,23,24]. Several meta-analyses have been performed to evaluate the inconsistency [25,26,27,28,29]. However, those meta-analyses focused on the associations between the total red meat intake and T2DM. Accumulating research suggests that red meat exerts an effect through its fat quantity and quality, branched-chain amino acids, heme iron content, and several substances produced during processing [30]. The Advanced Glycation End Products (AGEs) generated during the high-temperature making-processing and the nitrates–nitrites preservatives and salt added in processing may contribute to the different association of PRM and URM with health indicators (such as insulin resistance and abnormal glycemic profile) and multiplex diabetes risk factors [6,31,32,33]. A recent overview outlined that PRM have at least twice as high T2DM risk as URM [34]. Moreover, some recent studies indicated PRM and URM intake did not affect T2DM, which are not completely consistent with those previous meta-analyses [9,17,18,19].

Considering that the global red meat intake per capita is projected to double between the 1970s and 2030 [35], there is a clear need to clarify the equivocal findings and to elucidate potential sources of variation. Thus, the present meta-analysis was conducted to quantitatively assess the independent role of PRM and URM consumption in T2DM prevention.

## 2. Materials and Methods

This meta-analysis is reported in accordance with the Preferred Reporting Items for Systematic Reviews and Meta-Analysis (PRISMA) guidelines [36].

### 2.1. Data Source and Search Strategy

Electronic databases (PubMed, Embase, Web of Science, The Cochrane Library) were searched by two authors (R.Z. and J.L.F.) utilizing the following keywords: (meat OR red meat OR processed met) and (diabetes OR T2DM OR insulin OR insulin concentrations OR insulin resistance OR insulin sensitivity OR glucose OR fasting glucose). The reference lists of all identified trials and relevant reviews or editorials were also examined. The search was limited to English-language articles published between inception and January 2021.

### 2.2. Article Selection

Initially, article titles and abstracts were screened for relevance. The full text of potentially eligible articles was obtained to review eligibility for inclusion. The following criteria were used to select trials for inclusion in the review: (i) prospective cohort study; (ii) study explored the direct association between PRM or URM intake and T2DM; (iii) odds ratio (OR), relative risk (RR) or hazard ratio (HR) with their 95% confidence intervals (CIs) were provided. Selection criteria were not limited by study duration or participant demographics. Repeated publications for the same studies were excluded. Two researchers (R.Z. and J.L.F.) completed the study selection independently, and a third researcher (R.L.) adjudicated when there was disagreement pertaining to study inclusion

### 2.3. Data Extraction

Data extracted for each eligible trial included bibliographic information (author, publication year), participant characteristics, study follow-up duration, dietary assessment, outcome assessment, and effect sizes (OR, RR, and HR) with their corresponding 95% CIs. Extraction was performed by one researcher (R.Z.) and verified by a second researcher (J.L.F.).

### 2.4. Quality Assessment of Included Studies

Study quality was assessed using the Newcastle-Ottawa scale (NOS) [37], which includes three dimensions: selection of the study groups, comparability of the study groups, and outcome ascertainment and consists of eight questions with a minimum of zero and a maximum of nine points. Based on the number of points, studies were classified as: suboptimal quality (0–3), good quality (4–6), and excellent quality (7–9).

### 2.5. Statistical Analysis

Statistical analysis was performed using R software (R Foundation for Statistical Computing, Vienna, Austria), and graphing functions using GraphPad Prism 7 (GraphPad Software, California, America). The α level was set a priori for all statistical procedures at α = 0.05. RR with 95% CI being used as the measure of effect.

Fixed-effects meta-analysis was used where heterogeneity was low, and random effects meta-analysis was used for high heterogeneity [38,39]. Statistical heterogeneity was assessed using the Chi-square and I^2^ test [40], where a P-heterogeneity value < 0.10 and I^2^ > 50% was used to indicate high heterogeneity [41]. Sensitivity analyses were carried out by excluding one trial at a time to test the robustness of the pooled results [42]. Publication bias was evaluated by plotting standard errors against respective RR values, and then visually inspecting the funnel plot for symmetry. Funnel plot asymmetry was further assessed using Egger’s regression test [43]. Subgroup analyses was used to determine whether the following moderated effect size estimations: gender, geographical location, follow-up period, the number of participants and cases.

The method described by Orsini and Greenland et al. [44,45] were applied to explore the gender-specific dose–response relationship between PRM, URM and T2DM risk. Restricted cubic splines were calculated for each study with more than three categories of exposure, using three fixed knots at 10%, 50%, and 90% through the total distribution of the reported consumption of red meat, and combined them using multivariable meta-analysis. When studies reported only the total number of cases or total person-years and the exposure was defined in quantiles, the distribution of cases or person-years was calculated dividing the total number by the number of quantiles [29]. For studies that did not provide total person-years, person-years distribution was approximated from follow-up duration and number of participants [46].

## 3. Results

### 3.1. Literature Search and Trial Selection

**Figure 1:**
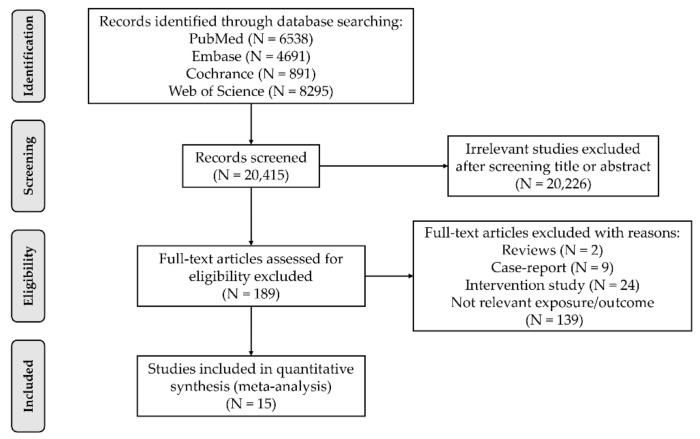
Flow chart of the study selection progress.

A total of 20,415 potentially eligible articles were identified. Following screening of abstracts and titles, 20,226 were excluded because they did not meet selection criteria. Of these, 15 prospective cohort studies were identified for inclusion. Reasons for exclusion included lack of relevant exposure/outcome (*n* = 139), intervention study (*n* = 24), case-report (*n* = 9), or review article (*n* = 2) (Figure 1).

### 3.2. Description of the Included Trials

A brief description of the included studies is given in Table A1. The follow-up duration ranged from 5 to 28 years. Seven studies (*n* = 465,995, age range = 25–75 years) investigated the relationship between PRM intake on T2DM risk (follow-up range = 5–28 y), of which 2 were conducted in Europe, 2 in the U.S., and 3 in Asia. Fourteen studies (*n* = 674345, age range = 20–90 y) investigated the relationship between URM intake and T2DM (follow-up range= 5–28 y), of which 7 were conducted in Europe, 4 in the U.S., and 3 in Asia. The quality of the included studies ranged from 7 to 8, with a median of 8.

### 3.3. PRM Consumption and T2DM Risk

PRM was associated with significantly larger RR for T2DM (RR: 1.27; 95% CI = 1.15–1.40); however, the heterogeneity was high (I^2^ = 81%). Two of the moderator variables were significant, sex (*p* < 0.01) and location (*p* < 0.01). For sex, the confidence interval crossed one when the sexes were combined (RR: 1.05; 95% CI = 0.97–1.14), but not when males and females were pooled independently. Further, the confidence intervals for males (RR: 1.41; 95% CI = 1.21–1.64) and females (RR: 1.30; 95% CI = 1.20–1.40) overlapped. For location, the confidence interval crossed zero for Asia (RR: 1.06; 95% CI = 0.98–1.14), but not Europe or the U.S. (Table 1).

### 3.4. URM Consumption and T2DM Risk

Compared with the lowest intake group, high consumption of URM increased T2DM incidence by 15% (RR: 1.15; 95% CI 1.08–1.23); however, the heterogeneity was high (I^2^ = 68%). Three of the moderator variables were significant, sex (*p* < 0.01), location (*p* = 0.01) and No. of cases (*p* = 0.02). For sex, the confidence interval crossed one when the sexes were combined (RR: 1.06; 95% CI = 0.99–1.14), but not when males and females were pooled independently. Further, the confidence intervals for males (RR: 1.24; 95% CI = 1.11–1.40) and females (RR: 1.13; 95% CI = 1.02–1.25) overlapped. For location, the confidence interval crossed one for Asia (RR: 1.10; 95% CI = 0.94–1.29) and Europe (RR: 1.08; 95%CI = 0.98–1.18), but not for the U.S. (Table 2).

### 3.5. Dose–Response Analysis Stratified by Sex

**Figure 2:**
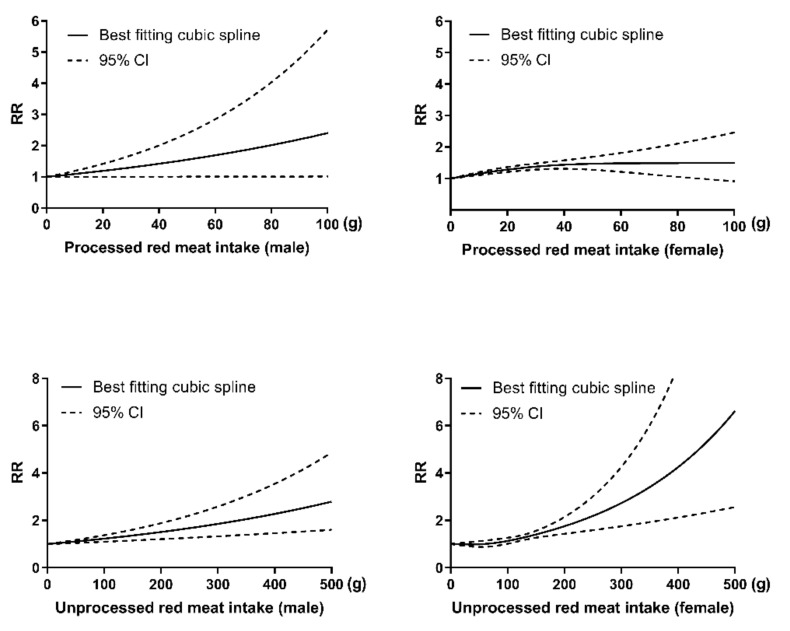
Dose–response relationship between consumption of PRM (male: P_linearity_ = 0.18; female: P_non-linearity_ < 0.01), URM (male: P_linearity_ = 0.40; female: P_non-linearity_ = 0.61) and risk of T2DM.

The analysis described above indicates that sex moderates the relationships between PRM and URM intake with T2DM risk. The risk of T2DM is increasing with the increased consumption of PRM and URM among both males and females, as shown in Figure 2.

### 3.6. Sensitivity Analysis and Publication Bias

**Figure 3:**
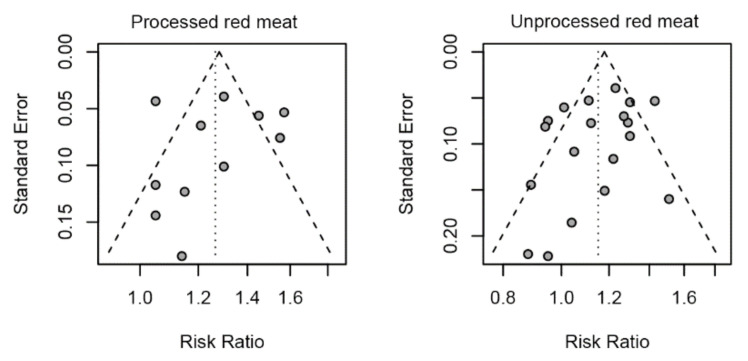
Funnel plot for association of TRM, PRM, URM with risk of T2DM.

Sensitivity analyses indicated that the pooled effects are robust. By omitting one study each time, sensitivity to each study is not found in our analysis. With respect to publication bias, visual inspection of the funnel plot did indicate asymmetry for PRM—there is a big gap to the right, but it did not reveal substantial asymmetry for URM. Egger’s regression test for funnel plot asymmetry yielded a nonsignificant result for PRM (*p* = 0.76) and URM (*p* = 0.19) (Figure 3).

## 4. Discussion

The associations between PRM and UPM consumption and risk of T2DM were evaluated in a meta-analyses of cohort studies. Compared with the lowest intake group, both high consumption of PRM (RR = 1.27, 95% CI = 1.15–1.40) and URM (RR = 1.15, 95% CI = 1.08–1.23) increased T2DM incidence. These relationships were moderated by location and sex. With respect to location, and across PRM and URM, the RR risk was consistently lowest for Asia-based studies and consistently highest for U.S.-based studies. Sex was a moderator for PRM and URM. However, when the sexes were analyzed separating the confidence intervals for males and females overlapped, indicating the effects of sex were inconclusive.

In our present study, red meat, both high consumption of PRM and URM, increased the odds of incidence of T2DM, which is in line with previous meta-analyses [6,35,47]. Before our study, the most recent published meta-analysis in 2013, which focused on the independent role of PRM and URM consumption in T2DM, found that high consumption of PRM increased T2DM incidence by 34%, and high consumption of URM increased T2DM incidence by 14% [47]. However, a recent large cohort study, involving 10,030 South Koreans aged 40–69 years in a 10-year follow-up, found no significant association between high PRM intake and incident T2DM [17], and several studies indicated that high URM intake did not increased the risk of T2DM [13,18,19]. Thus, we updated a meta-analysis to provide the most up-to-date information on this topic and results from the latest meta-analysis showed that high consumption of PRM and URM were associated with 27% and 15% higher risk of T2DM, respectively. There are some plausible biological mechanisms that may help explain the relationship between red meat consumption and T2DM risk. First, red meat is generally high in saturated fats and branched-chain amino acids, which may subsequently result in increased serum-free fatty acids and lead to insulin resistance both in liver and muscle [34]. Second, serum ferritin and glycine concentrations are both associated with insulin resistance and oxidative stress, which was proposed as a potential mechanism [48,49]. Third, heme iron, provided mainly by red meat intake, can promote free fatty acid oxidation and increase levels of free radicals, which subsequently damage the beta cells of the pancreas [7]. Additionally, the association between red meat and diabetes may be in part confounded by the indirect effect of obesity and hyperuricemia [21,50].

We also found that the confidence interval for PRM (RR = 1.27, 95% CI = 1.15–1.40) is larger than URM (RR = 1.15, 95% CI = 1.08–1.23), although they were not directly compared, which could suggest that PRM may have a higher risk of diabetes, which is consistent with previous studies [6,47]. In 2010, a meta-analysis documented 19% higher diabetes risk per 50 g/day PRM, while URM does not reach significance [28]. Additionally, a study reported that replacing PRM with UPM reduced T2DM incidence (HR 0.96, 95% CI 0.93, 0.99) [51]. Factors which might help to explain the higher RR for PRM include higher saturated fatty acid, sodium, heme iron, and salt content [28,52,53], the aromatic hydrocarbons, heterocyclic amines and Advanced Glycation End Products (AGEs) that arise from high-temperature making–processing [54,55] and the nitrates–nitrites preservatives added in processing [52].

The heterogeneity for the associations between PRM and URM with T2DM were high. Moderator analysis revealed that location and sex explained some of the heterogeneity. In particular, for all types of red meat consumption, RRs were consistently highest for U.S.-based studies, and consistently lowest for Asian-based studies. An important consideration when interpreting this observation is the variation in red meat consumption. Compared with Europe and the U.S., Asian populations have lower red meat intake [17,22,30,56]. It is possible that relatively low red meat intake is not enough to increase T2DM incidence. For each type of red meat consumption, the confidence intervals crossed one when, combined, the sex and the confidence intervals for males and females overlapped when the sexes were analyzed separating. As such, the effects of sex are inconclusive. Additional studies are required to test for sex differences in the relationship between PRM, URM and T2DM.

Reduction of red meat consumption should be explored as a target for T2DM prevention initiatives. Such initiatives are likely to be most effective in the U.S., where red meat intake is high. One study conducted in 2019 reported that T2DM incidence decreased when red meat was substituted with poultry (HR: 0.96, 95% CI 0.93, 0.99) or fish (HR: 0.94, 95% CI 0.91, 0.97) [51], and, recently, a cohort study including 148,853 participants found that replacing red meat consumption (both PRM and URM) with other protein sources (poultry, seafood, egg, legumes and nuts) was associated with a lower risk of T2DM, and stronger for the replacement of PRM [57]. Further research is demanded to explore the effect of a decreased intake of red meat and simultaneous increased intake of other high-protein foods on diabetes.

The major strengths of our research include a large sample size (*n* = 682,963 participants, cases = 50,675), high quality of included trials, robust effect sizes, moderator analysis to explain potential sources of heterogeneity, low potential of potential bias and dose–response analysis. Besides, our study was the latest meta-analysis focusing on the independent role of PRM and URM consumption in T2DM. However, there are several potential limitations that should be considered when considering the findings of the current meta-analysis. First, the included studies were based on observational prospective research designs, which are prone to confounding. Most of the studies did adjust for the most important confounding factors, including BMI (100%), physical activity and energy intake (93%), and education and smoking (87%). However, only 53.3% adjusted for family history of diabetes and 40% adjusted for the history of chronic diseases. Second, some studies only assessed dietary intake at baseline, and red meat consumption change over time was not assessed. In the three Harvard cohorts, increased and decreased red meat intakes were found to be associated with higher and lower risk of T2DM, respectively [47]. Third, the follow-up time span was generally large among the included studies, which may result in temporal bias. Last, the method of determining T2DM cases differed between studies, with five studies relying on self-report.

## 5. Conclusions

Compared with the lowest intake group, both high consumption of PRM (RR = 1.27, 95% CI = 1.15–1.40) and URM (RR = 1.15, 95% CI = 1.08–1.23) increased T2DM incidence. These relationships were consistently strongest for U.S.-based studies, though the effects of sex are inconclusive. Reduction of red meat consumption should be explored as a target for T2DM prevention initiatives.

## Figures and Tables

**Table 1:** The association between PRM intake and risk of T2DM.

Subgroup	No. of Included Studies	RR (95% CI)	*p* Value	I^2^
Overall	7	1.27 (1.15, 1.40)		81.40%
Gender			<0.01	
Both	2	1.05 (0.97, 1.14)		0.00%
Male	4	1.41 (1.21, 1.64)		61.90%
Female	5	1.30 (1.20, 1.40)		44.10%
Location			<0.01	
Europe	2	1.26 (1.06, 1.50)		0.00%
US	2	1.40 (1.28, 1.55)		73.90%
Asia	3	1.06 (0.98, 1.14)		0.00%
Follow-up			0.15	
<10 years	1	1.11 (0.92, 1.33)		0.00%
≥10 years	6	1.29 (1.16, 1.44)		86.10%
Sample size			0.10	
<10,000	2	1.08 (0.89, 1.30)		0.00%
≥10,000	3	1.29 (1.16, 1.44)		84.10%
No. of Case			0.47	
<5000	4	1.23 (1.09, 1.38)		53.20%
≥5000	3	1.32 (1.12, 1.57)		92.60%

**Table 2:** The association between URM intake and risk of T2DM.

Subgroup	No. of Included Studies	RR (95% CI)	*p* Value	I^2^
Overall	14	1.15 (1.08, 1.23)		67.90%
Gender			<0.01	
Both	5	1.06 (0.99, 1.14)		0.00%
Male	7	1.24 (1.11, 1.40)		60.40%
Female	7	1.13 (1.02, 1.25)		73.20%
Location			0.01	
Europe	7	1.08 (0.98, 1.18)		41.50%
US	4	1.27 (1.18, 1.36)		39.70%
Asia	3	1.10 (0.94, 1.29)		60.60%
Follow-up			0.47	
<10 years	3	1.07 (0.84, 1.35)		59.90%
≥10 years	11	1.17 (1.09, 1.25)		67.70%
Sample size			0.12	
<10,000	3	0.99 (0.82, 1.20)		8.40%
≥10,000	11	1.17 (1.09, 1.25)		70.20%
No. of Case			0.02	
<5000	10	1.09 (0.99, 1.19)		55.10%
≥5000	4	1.25 (1.15, 1.35)		65.00%

## Data Availability

Data are contained within the article.

## Appendix A

**Table A1:** Characteristics of included studies.

Author	Country/ Published Year	Follow-up Period	Age at Entry	Gender	Study Cases /Size	Case Ascertainment	Ascertainment of T2DM	Adjusted Factors	NOS Score
**PRM**									
Kurotani et al.	Japan/2013	5	45–75	both	1178/63,849	self-reported	History of clinical diagnosis	1,5–11,18,19,23, 27,29,31–34	8
Lajous et al.	France/2012	20	NA	female	1369/66,118	self-reported and medical records	History of clinical diagnosis or reimbursement claims for diabetes medications.	3,5,6,8,10–12,18–22, 24,29,30,42	7
Pan-HPFS et al.	US/2011	20	40–75	male	2438/37,083	self-reported and medical records	History of clinical diagnosis or National Diabetes Data Group criteria	1,5–9,11,14,16, 19,20,39	8
Pan-NHS1 et al.	US/2011	28	30–55	female	8253/79,570	self-reported and medical records	History of clinical diagnosis or National Diabetes Data Group criteria	1,5–9,11,14,16, 19,20,39	8
Pan-NHS2 et al.	US/2011	16	25–42	female	3068/87,504	self-reported and medical records	History of clinical diagnosis or National Diabetes Data Group criteria	1,5–9,11,14,16, 19,20,39	8
Son et al.	Korean/2018	10	40–69	both	668/8618	self-reported	History of clinical diagnosis, currently receiving medication, or use of insulin or oral medication	1–3,5–10,12,13, 18,23,30,35,40	7
Steinbrecher et al.	US/2011	14	45–75	both	8587/75,512	self-reported and medical records	History of clinical diagnosis or currently receiving medication	3,5,8,9,14	8
Talaei et al.	Singapore/2017	18	45–74	both	5027/45,411	self-reported	History of clinical diagnosis	1,3,5–9,13,15,19, 39,50,51	7
Virtanen et al.	Finnish/2017	19.3	42–60	male	432/2330	self-reported, medical records and examination	History of clinical diagnosis, fasting plasma glucose ≥ 7.0 mmol/L or 2 h oral glucose ≥ 11.1 mmol/L	1–12,17,21,25,27,29, 30,40,41,51,59–63	8
**URM**									
EPIC-InterAct et al.	Europe/2013	11.7	20–80	both	11,559/26,088	self-reported and medical records	History of clinical diagnosis or currently receiving medication	3,5–9	8
Ericson et al.	Sweden/2015	14	45–74	both	2860/26,930	registry and examination	Fasting plasma glucose concentration ≥7.0 mmol/L or fasting whole blood concentration ≥ 6.1 mmol/L	1,3,5–9,13,52,56	7
Fretts et al.	US/2012	5	18–75	both	243/2001	examination	American Diabetes Association’s criteria	1,3,5–9,11–13, 18,36	8
Kurotani et al.	Japan/2013	5	45–75	both	1178/63,849	self-reported	History of clinical diagnosis	1,5–11,18,19, 23,27,29,31–34	8
Lajous et al.	France/2012	20	NA	female	1369/66,118	self-reported	History of clinical diagnosis or reimbursement claims for diabetes medications.	3,5,6,8,10–12, 18–22,24,29,30,42	7
Mannisto et al.	Finland/2010	12	50−69	male	1098/25,943	medical records	History of clinical diagnosis	1,5–10,26,29,30, 37,38,41,57	7
Mari-Sanchis et al.	Spain/2016	14	20−90	both	146/18,527	self-reported and medical records	History of clinical diagnosis or currently receiving medication	1,5,6,8,9,11–13, 19,20,24,25,39,	8
Pan-HPFS et al.	US/2011	20	40–75	male	2438/37,083	self-reported and medical records	History of clinical diagnosis or National Diabetes Data Group criteria	1,5–9,11,14,16, 19,20,39	8
Pan-NHS1 et al.	US/2011	28	30−55	female	8253/79,570	self-reported and medical records	History of clinical diagnosis or National Diabetes Data Group criteria	1,5–9,11,14,16, 19,20,39	8
Pan-NHS2 et al.	US/2011	16	25−42	female	3068/87,504	self-reported and medical records	History of clinical diagnosis or National Diabetes Data Group criteria	1,5–9,11,14,16, 19,20,39	8
Steinbrecher et al.	US/2011	14	45–75	both	8587/75,512	self-reported and medical records	History of clinical diagnosis or currently receiving medication	3,5,8,9,14	8
Talaei et al.	Singapore/2017	18	45–74	both	5027/45,411	self-reported	History of clinical diagnosis	1,3,5–9,13,15,19, 39,50,51	7
Van et al.	Dutch/2012	13	≥55	both	456/4366	registry	History of clinical diagnosis or currently receiving medication	1,5–7,9,11–13,23,24, 34,38,42,43,46–48	7
Villegas et al.	China/2006	5	40–70	female	1972/70,609	self-reported	History of clinical diagnosis or fasting glucose ≥ 7 mmol/L on at least two occasions or an oral glucose tolerance test ≥ 11.1 mmol/L and/or currently receiving medication	1,2,3,5–10,19, 28,45	7
Virtanen et al.	Finnish/2017	19.3	42–60	male	432/2330	self-reported, medical records and examination	History of clinical diagnosis, fasting plasma glucose ≥ 7.0 mmol/L or 2-h oral glucose ≥ 11.1 mmol/L	1–12,17,21,25,27,29, 30,40,41,51,59–63	8
Van Dam et al.	America/2002	12	40–75	male	1321/42,504	self-reported, medical records and examination	An elevated plasma glucose concentration plus at least one classic symptom or at least two elevated plasma glucose concentrations on different occasions or treatment with insulin or oral hypoglycemic medication	1–9,11,17,26, 27,51,58	8

T2DM: Type II diabetes mellitus; NOS: Newcastle-Ottawa scale; PRM: processed red meat; URM: unprocessed red meat; 1: age; 2: income; 3: education; 4: marital status; 5: BMI; 6: smoking; 7: alcohol use; 8: physical activity; 9: energy intake; 10: vegetable intake; 11: family history of diabetes; 12: fiber intake; 13: sex; 14: ethnicity; 15: heme iron intake; 16: menopausal status and hormone use in women; 17: serum ferritin; 18: geographic area; 19: history of chronic diseases; 20: hypercholesterolemia; 21: polyunsaturated fatty acid; 22: hormone replacement therapy; 23: fat intake; 24: carbohydrates; 25: glycemic index; 26: blood pressure; 27: magnesium; 28: WHR; 29: coffee; 30: fruit; 31: Calcium; 32: soft drink consumption; 33: rice intake; 34: fish intake; 35: sodium; 36: glycemic load; 37: rye; 38: milk; 39: diet adherence; 40: medication use; 41: serum cholesterol; 42: unprocessed red meat; 43: tea; 44: caffeine intake; 45: occupation status; 46: cheese; 47: poultry; 48: soya; 49: snacking; 50: dialect; 51: year; 52: season; 53: multiple pregnancy; 54: parity; 55: special diet; 56: method version; 57: intervention group; 58: whole grain; 59: berries; 60: saturated fatty acid; 61: monounsaturated fatty acid; 62: fasting plasma glucose; 63: fasting serum insulin.
